# Cardiovascular risk management in patients using antipsychotics: a qualitative feasibility study

**DOI:** 10.3399/BJGPO.2025.0014

**Published:** 2026-01-14

**Authors:** Karlijn Joëlle van den Brule-Barnhoorn, Kirstine Marieke Jakobs, Jan van Lieshout, Sietske Grol, Joost Janzing, Wiepke Cahn, Marion Biermans, Erik Bischoff

**Affiliations:** 1 Department of Primary and Community Care, Radboudumc, Nijmegen, Netherlands; 2 Zorggroep Onze Huisartsen, Arnhem, Netherlands; 3 IQ Health Science Department, Radboudumc, Nijmegen, Netherlands; 4 Department of Psychiatry, Radboudumc, Nijmegen, Netherlands; 5 Department of Psychiatry, UMC Utrecht, Utrecht, Netherlands; 6 Department of General Practice, Erasmus MC, Rotterdam, Netherlands

**Keywords:** antipsychotic agents, feasibility, heart diseases, medication review

## Abstract

**Background:**

Patients on antipsychotic medication (APM) have an increased risk of cardiovascular disease (CVD). In general practice, however, there is a lack of solid cardiovascular risk (CVR) management for this specific group. TACTIC, a person-centred multidisciplinary cardiovascular risk programme, which aims to decrease cardiovascular risk and review APM use, was piloted in general practice.

**Aim:**

To explore barriers and facilitators for delivering the TACTIC intervention, and assess which adjustments have to be made to evaluate its effectiveness and implementability in a future randomised controlled trial (RCT).

**Design & setting:**

Qualitative analysis of the feasibility study in three Dutch general practices.

**Method:**

We performed eight individual interviews with patients and two focus group interviews with 11 healthcare professionals (HCPs) involved in the study. Interviews were semi-structured and topic guides were informed by the normalisation process theory (NPT). We used the framework method for analysis of our data.

**Results:**

The three main barriers for delivering TACTIC were as follows: the tension towards the intervention experienced by patients; the course of the multidisciplinary meeting (MDM); and the high workload experienced by GPs. The main facilitators were associated with the person-centred approach, the clear information meeting, and the ability of adjusting roles during the course of the intervention, including bringing a carer. Valuable suggestions for improvement were introducing a summary report from the psychiatrist, improving expectation management for patients, and adjusting the definition of the target group.

**Conclusion:**

Several adjustments to the TACTIC intervention are necessary before evaluation in a larger RCT can take place. This work underlines the importance of performing a feasibility study before a trial to improve its effectiveness and efficacy.

## How this fits in

Cardiovascular risk management, although common in general practice, is insufficiently applied to patients on antipsychotic medication (APM), despite the knowledge that this patient group is at increased risk of developing cardiovascular disease (CVD). We developed TACTIC, a person-centred multidisciplinary intervention aimed at decreasing cardiovascular risk and reviewing medication use, and implemented it in three general practices. With this feasibility study, we assessed the experiences of patients and healthcare professionals (HCPs), and explored the barriers and facilitators for delivering the TACTIC intervention, together with suggestions for improvement. The findings highlight the valuation of a person-centred approach in relatively vulnerable patients, and underscore the importance of good expectation management and defining the appropriate target group in order for the intervention to succeed.

## Introduction

Antipsychotic medication (APM) is used in general practice for treatment of a range of disorders. Primarily, it is indicated for severe mental illnesses (SMIs), including schizophrenia, bipolar disorders, and affective psychoses, and the medication is found to be effective.^1–3^ APM is prescribed to 1–2% of the general population.^4,5^ A large proportion of antipsychotics, however, ranging from 35–77%, is prescribed off-label, for treatment of anxiety, dementia, and sleep and personality disorders.^4,6–11^ The use of atypical (or second-generation) APM is increasing worldwide,^6,12–16^ especially as a consequence of increased off-label use.^6,17–20^

This raises concerns about the risk of severe side effects.^6^ Use of APM is associated with an increased risk of cardiovascular events in 29% of women and in 15% of men.^21^ This is owing to metabolic effects, such as glucose intolerance, dyslipidaemia, weight gain,^22^ and hypertension.^23^

Even though the evidence on the increased cardiovascular risk (CVR) is well established, monitoring of patients is insufficient.^24–29^ The clinical guideline for CVR management of the Dutch College of General Practitioners (NHG) does not even mention the use of APM as a risk factor.^30^ In September 2024, an update of the guideline was published, only advising GPs to consider drawing up a risk profile for patients with an SMI.^31^

GPs are generally less aware of the side effects of APM than psychiatrists,^32^ which contributes to a lack of solid follow-up.^33^ Unfortunately, GPs and psychiatrists often do not collaborate, even when it comes to reducing CVR in patients using APM.^33^ Care involving collaboration and coordination between different levels of care, such as primary and secondary care, may have added value in reducing CVR in patients taking APM. In the Netherlands, this type of care is called ‘transmural’ collaborative care.

Together with a multidisciplinary task force, consisting of relevant stakeholders in the Eastern Netherlands,^34^ we developed a person-centred intervention to address these problems. Our intervention, called TACTIC, covers Transmural collaborative care regarding CVR management and medication review for patients using AntipsyChoTICs.^35^ With this intervention, GPs closely collaborate with patients, psychiatrists, and other disciplines to reduce CVR and review APM use. Based on advice with psychiatrist’s input, future steps are planned in a shared decision-making process by GP and patient. TACTIC is considered a complex intervention, as defined by the British Medical Research Council, both owing to the structure of the intervention, as well as the complexity that arises from the interaction between the intervention and the context in which it is implemented.^36^

### Aim

The purpose of this qualitative feasibility study was to assess the acceptability of the procedures of TACTIC and to explore barriers and facilitators for delivering the intervention. The results will provide suggestions for improvement in order to evaluate TACTIC’s effectiveness and implementability in a future randomised controlled trial (RCT).

## Method

### Study design

**Figure 1.**
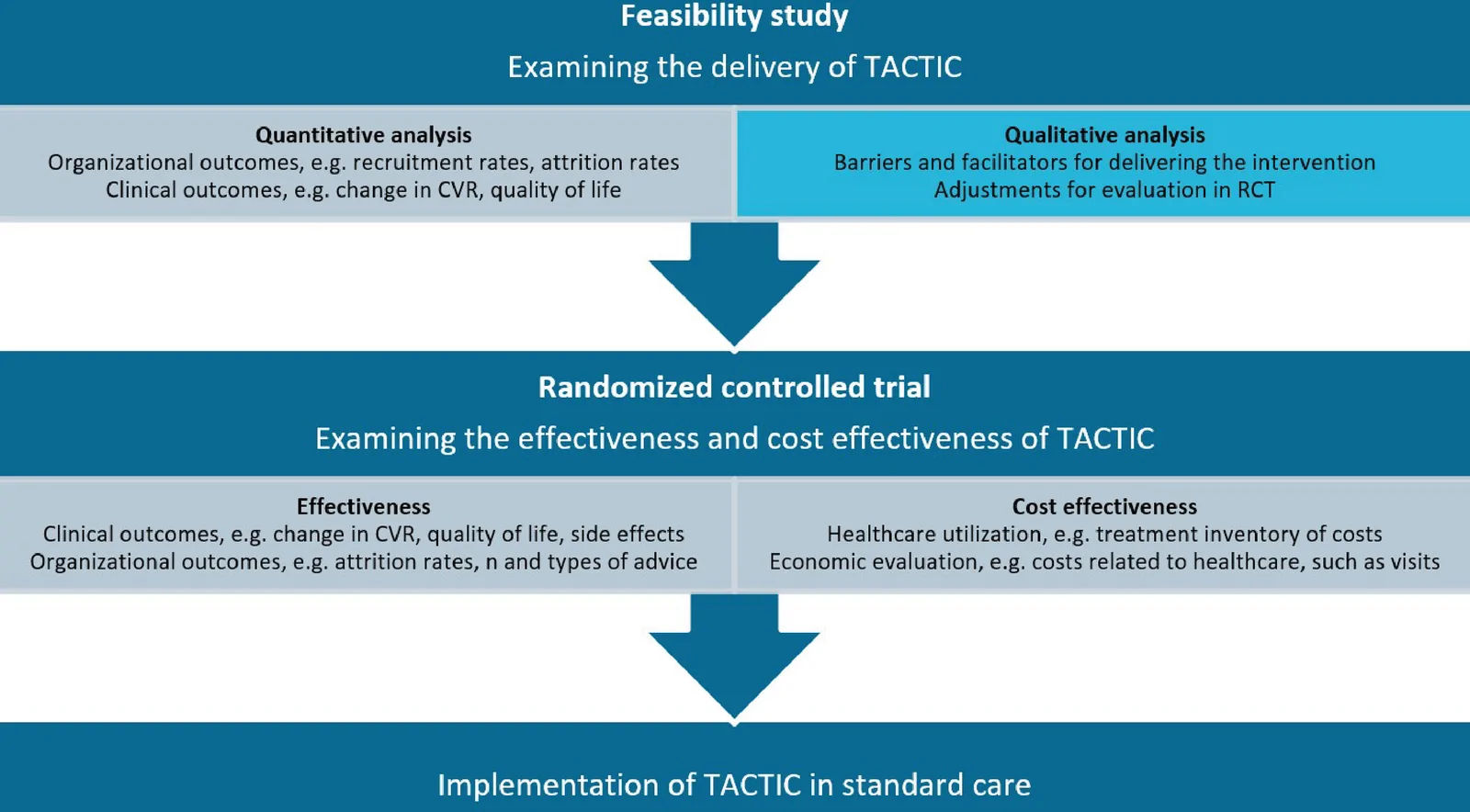
TACTIC project overview with the current qualitaive part of the feasibility study highlighted in bright blue. CVR = cardiovascular risk. RCT = randomised controlled trial

This qualitative study is part of a larger TACTIC project, see Figure 1. As TACTIC is considered a complex intervention, it is recommended to perform a mixed-methods feasibility study before conducting a trial.^36^ In line with other studies, we use the term feasibility study as an overarching term for studies that aim to support the development of future studies.^37,38^

Methods and results of the qualitative part of the feasibility study are presented in the current article. More detailed information on the quantitative part, including patient selection and study flow, is published elsewhere.^35^

### Setting and participants

The feasibility study took place in 2021. We implemented TACTIC in three general practices in an urbanised region in the Eastern Netherlands at the same time, and followed patients for 3 months.

TACTIC is aimed at patients using APM for at least 3 months, prescribed by their GP, who have no psychiatrist involved in their current treatment phase. Patients were excluded from participation if they were aged <25 years or >84 years, had a diagnosis of dementia or organic psychosis, or had a history of cardiovascular disease (CVD).

GPs generated a list of eligible patients from the electronic medical records, based on inclusion and exclusion criteria, and subsequently invited the selected patients by telephone. Ultimately, 28 patients participated. The intervention was completed by 23 patients, of whom 18 had a complete follow-up. At baseline, dropouts did not differ from the participants who completed follow-up.^35^

### Intervention

**Figure 2.**
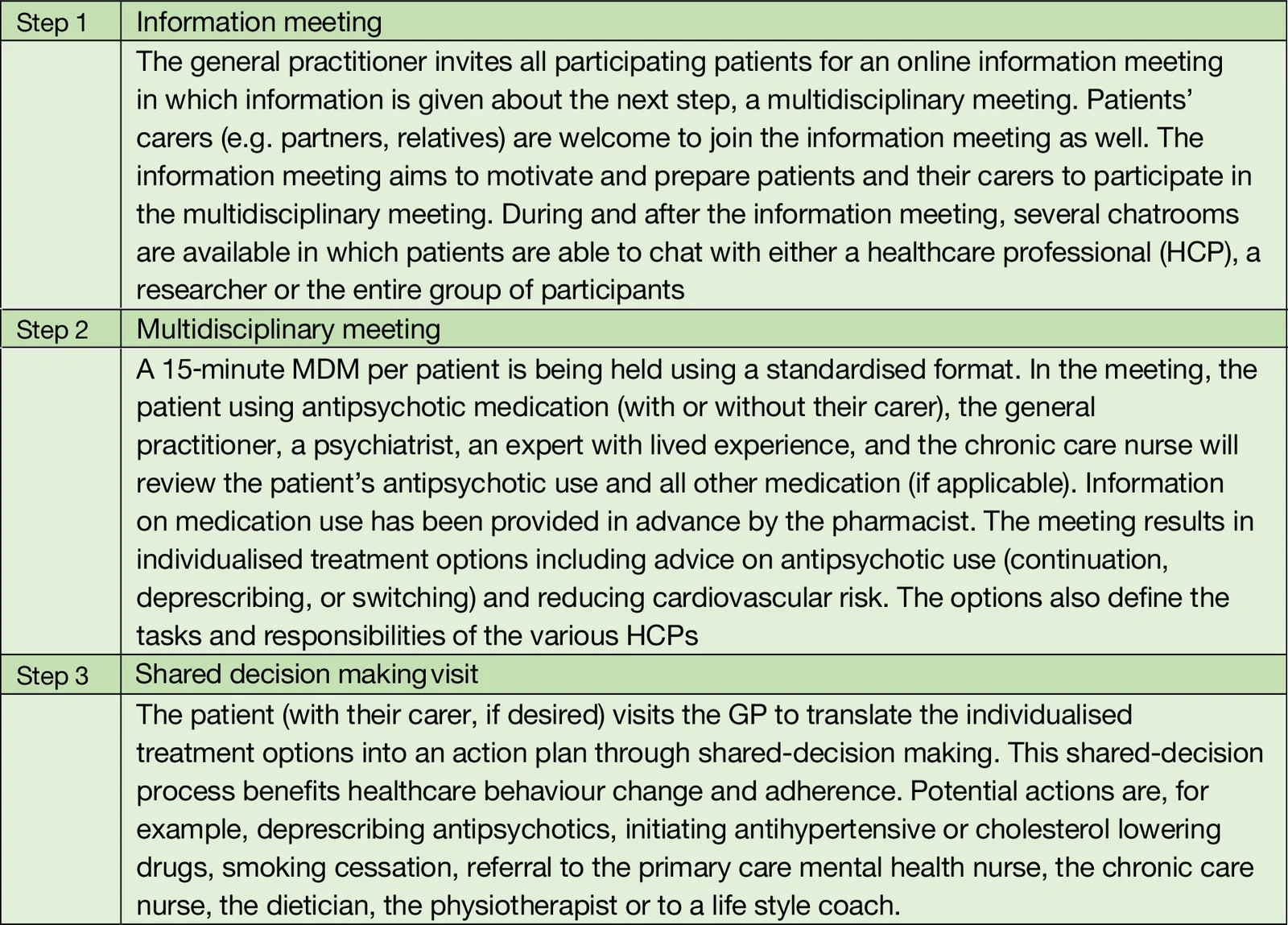
TACTIC intervention. HCP = healthcare professional. MDM = multidisciplinary meeting.

TACTIC consists of the following three consecutive steps: an information meeting; a multidisciplinary meeting (MDM); and a shared decision-making (SDM) visit with the GP (Figure 2) Patients are welcomed and encouraged to bring a carer throughout the intervention to provide social support, as this can be an important motivating factor.^39,40^

### Data collection

Four months after implementation of TACTIC in the three general practices, we performed individual interviews with participating patients and their carers. Two focus group interviews were held with healthcare professionals (HCPs) involved in the feasibility study.

Owing to the delicate subject of the study, we chose to interview patients individually, to ensure a safe environment in which they could speak openly about their personal experiences with TACTIC.

During the focus group meetings, however, we used the group dynamic and interaction among HCPs to help explore the barriers and facilitators in conducting TACTIC in-depth.

For the individual patient interviews, we purposively sampled patients who completed the entire intervention, based on CVR, sex, age and on- or off-label use of APM, to make sure all relevant characteristics were represented. For the focus group interviews, we invited all HCPs involved in the study.

All participants received oral and written information about the study and its aims, and were subsequently invited to participate.

KB conducted the individual interviews. The interviews took place at the patient’s home, their working place, their general practice, online through video call, or by telephone, whichever was convenient for the patient. SG moderated the focus group interviews, and KB and KJ attended as observers. Owing to the COVID-19 pandemic, we had to organise the focus groups digitally through video call instead of in real life.

We audiorecorded all interviews after obtaining informed consent. To protect the participants’ identities, ID codes were used throughout data handling.

The individual and focus group interviews were semi-structured. The topic guides were informed by the four constructs of the normalisation process theory (NPT) (Supplementary Box 1).^41,42^ Questions focused on key trial parameters, addressing components such as sense-making, workability, enrolment, appraisal, and reconfiguration (Supplementary Tables 1 and 2). The guides were partly adapted to each participant category and initial findings influenced sequential topic guides to assure we collected all necessary data to answer our research questions.

We applied the Standards for Reporting Qualitative Research (SRQR).^43^

### Data analysis

We used the framework method for the analysis of our data (Supplementary Box 2).^44^ All interviews were transcribed verbatim and, after familiarisation with the data, coded using Atlas.ti (version 24.0.0). The first three individual interviews were coded by KB and KJ independently, after which they agreed on a set of codes to apply to all subsequent transcripts, which were then coded by KB. The focus group interviews were coded by KB and KJ independently, and after review with the research group they reached consensus.

We coded the data inductively using a thematic analysis. The NPT proved to be less helpful than anticipated and using NPT would mean forcing the data into the predetermined constructs. For this reason, we restricted the use of the NPT to the development of the interview guide and abandoned its use in the analyses. Consequently, codes were grouped into clearly defined categories by the research group, to form a working analytical framework. After applying the analytical framework to all transcripts, data were charted into a framework matrix and interpreted for analysis.

Finally, the project team discussed the lessons of the feasibility study during and after the pilot phase in order to embed them in the effectiveness study.

## Results

We conducted eight individual interviews with patients and two focus group interviews with 11 out of 13 HCPs in total (two pharmacists cancelled last minute, of whom one provided written input). During one individual interview, the patient’s partner was also present. See Tables 1 and 2 for participant characteristics.

**Table 1.** Participant characteristics individual interviews

Participant	Interview time, minutes	Risk of CVD, % QRISK3	M or F sex	Age, years	APM-use, on or off-label
P02	50	10.6	M	54	On-label
P03	40	5.0	M	44	Off-label
P04	41	54.0	F	55	Off-label
P06+C06	27	19.9	M	68	Off-label
P07	31	0.5	F	27	Off-label
P15	33	3.0	F	46	Off-label
P17	45	5.2	M	51	Off-label
P25	55	9.1	M	45	On-label

APM = atypical antipsychotic medication. C = carer. CVD = cardiovascular disease. P = patient. QRISK3 = 10-year risk of cardiovascular disease

**Table 2.** Participant characteristics focus group interviews

Focus group session	Participant	Occupation	General practice
(1) 1 hour and 23 minutes	CN1	Chronic care nurse	1
	EE2	Expert with lived experience	1
	GP1	GP	1
	GP2	GP	2
	GP3	GP	3
	Ph1^a^	Pharmacist	1
	Ps	Psychiatrist	1, 2 and 3
(2) 1 hour and 24 minutes	CN2	Chronic care nurse	2 and 3
	EE1	Expert with lived experience	2 and 3
	GP4	GP	1
	GP5	GP	2
	Ph2	Pharmacist	1

^a^Provided written input. CN = chronic care nurse. EE = expert with lived experience. Ph = pharmacist. Ps = psychiatrist

After six individual interviews, we presumed data saturation, after which two more interviews were performed to check and confirm data saturation.

During familiarisation and refining the framework, five main themes were identified. These were as follows: goal of TACTIC; expectations of the intervention; experiences and feelings; communication and information; and the role of HCPs (Table 3).

**Table 3.** Themes and representative quotes related to TACTIC

Theme	Representative quote
**Goal of TACTIC**	*‘What I do like is that there is structural attention to the possible negative side effects of medication*.’ (P03)
	*‘Taking antipsychotics can pose health risks. By no means everyone knows this and for a long time it was not monitored either. So I'm glad that was done now, even with me as someone taking low-dose antipsychotics prescribed by my GP*.’ (P25)
	*‘Before you go into the whole process, hey, if it’s going to become a treatment method, estimate very carefully whether it really adds value for the patient in question*.’ (P03)
	*‘If indeed you already have people who* [...] *have low quetiapine use and are otherwise hardly at increased cardiovascular risk, you can cozy up to them and you can look at whether or not it’s smart to continue using the drug, but when you say: how much health gain you're making, it’s very limited*.’ (Ps)
	*‘I mean: you don't aim for those people to stop taking pills, do you? You aim for those people to be stable and somewhat satisfied that they have all reasonable values, and you look at where you can make some adjustments, you adjust there. That’s one, in my opinion. And you have those people who do have a poor risk profile, you want to adjust that. Those are your objectives, I think, briefly*.’ (GP3)
	*‘Not, for example, revising the diagnosis or turning your whole medication policy upside down. Of course, suggestions can be made, but if that is too complex, it does not belong in general practice. Then you would have to refer that back to the psychiatrist again*.’ (GP4)
**Expectations of the intervention**	*‘I, um, was dealing with some issues myself about medication and side effects. And um, yeah, so that actually came at a good time*.’ (P02)
	*‘I basically had to explain my own situation and then we got some general tips back. So that was very disappointing for me. I get that they can't all look deep into the file. I understand that too, but the idea behind it seemed very good to me. So that you talk to a number of people: are there other routes possible, other than just with your GP? But you really need more time for that and perhaps people need to have studied your file a little more, I think, if you really want such a group discussion to succeed*.’ (P17)
	*‘… maybe check at the beginning: this is what we want to talk about, what do you want to talk about? Otherwise there will be another one of those jack-in-the-box things at the end*.’ (GP5)
**Experiences and feelings**	*‘I found it very stressful to participate. Because yes that ... It is drastic in your life when you are going to change something. I found that very stressful*.’ (P06) *‘He actually experienced it all very emotionally. From his own fear of: I can’t do without antipsychotics*.’ (C06)
	*‘A lot of people who use for a long time, they've been told: you have a lifelong condition or illness and you have to take medication for the rest of your life. And if you’re going to phase that out then people might be afraid, because then there’s going to be a crisis, and the care providers might also be afraid*.’ (P25)
	*‘I think they might underestimate it a bit how stressful it is for participants. And certainly people who have been dependent on the drug for a long time. And that a bit more reassurance at the very beginning of that process is therefore necessary*.’ (P06)
	*‘I very much felt that I had to defend my own point of view and stuff. And that the psychiatrist very much had the idea that I should want to taper or reduce and that the feeling came up very much like: that’s the whole idea behind the study, that you just stop. Yes, ho, sorry, we're not going to do it like that*.’ (P07)
	*‘That* [GP] *said: yes, we can also just watch it now and if later on you do feel the need to ... to taper off or that you think it’s necessary, yes, then we can still talk about it*.’ (P07)
	*‘I found it hard to stay motivated myself, to just keep participating throughout the study*.’ (GP4)
	*‘When you talk about selection etc., it’s about the goal you're aiming for. Are you aiming mainly to start seeing or working with patients who have an increased cardiovascular risk* [...] *then you could say: that is the category that is most relevant to look at critically*.’ (Ps)
**Communication and information**	*‘It made me feel like I was really participating in something. And that’s different than just getting mail*.’ (P04)
	*‘I did think it made sense that each discipline, each person involved briefly told who they were and what they were doing and what to expect. I think it was functional to know a little bit about who was involved*.’ (GP4)
‘	*‘What I can imagine is just some kind of standard video in terms of, say, the people participating in this project*.’ (Ps)
	*‘And those cardiovascular diseases didn’t actually come out er … clearly. And that … yes, if you look at it in retrospect, that could have been a bit clearer*.’ (P02)
	*‘… people might have to read up on you just a little bit more, I think, for such a meeting to really succeed*.’ (P17)
	*‘I read them all, also as a piece of preparation when people came to me after 3 months. I did enjoy reading them. I also put them neatly in the medical record, absolutely great.’* (CN1)
**The role of HCPs**	*‘It’s the energy it took and the time it took and the yield, those were not feasible for me either*.’ (GP5)
	*‘If the GP calls themselves, that does help, yes. That’s pretty labour intensive, I do think, for the GP in this case, but it is the most effective way to include patients, I think*.’ (GP1)
	*‘I think it does require a certain level of trust and you can't just let a random practice assistant make a call*.’ (GP2)
	*‘Multiple perspectives might help us forward. Yes, if that is done very openly, I think that is the right start.*’ (P17)
	*‘Well, what I really liked about that is that there was room to indicate things*. […] *I had indicated: I don't want the expert with lived experience to be there. And that there was the possibility, that this was considered, of... yes, "that’s possible! That can be arranged."’* (P07)
	*‘There was not really an equal dialogue partnership. All of us could pay more attention to that, I think*.’ (GP5)
	*‘Because then I also hear the stories from the patients and then I can build on that during the following consultation, or I think: ”oh yes, he can work with that, or that was the psychiatrist’s suggestion*.”’ (CN1)
	*‘The presence and input of the expert with lived experience, for me it was a surprise how valuable and meaningful that was*.’ (GP4)
	*‘I think the expert with lived experience, she wasn’t — she didn’t add anything for me, because she was coming from a completely different situation.’* (P17)
	*‘The moment you would add experiential expertise of a good quality to the palette of your general practice, then it also falls much more into place.*’ (EE1)
	*‘If I hadn't been there he wouldn't have ... he would have dropped out.’* (C06) *‘I would have dropped out then, yes.’* (P06)
	*‘At this stage of my rehabilitation, yes, I can largely provide myself with everything I need. In another phase, it might have been nice, if it were 10 years back, or 15 years back.*’ (P25)
	*‘Now it was mostly a paper session, so you just look through the medication status and the tally sheet with the reported side effects, you link it together and you comment on that and you give the advice to taper off, but yes, if you don't know the patient or you have no additional information, then sometimes I know in advance that tapering off is really not possible and so it’s useless advice*.’ (Ph2)
	*‘The advice of a pharmacist may contribute to good compliance and possibly also increase client involvement in treatment, and thus the success of the treatment*.’ (Ph1)
	*‘If you think for instance: add another pharmacist, then it will only get busier and I don't think the patients would like that at all, because then there would be even more people they don't know. That’s almost more threatening, I think.*’ (GP3)

HCP = healthcare professional

### Goal of TACTIC

Patients saw TACTIC as a relevant health check. They appreciated the attention to the metabolic effects of their treatment. Both patients and HCPs saw TACTIC as a suitable way to create more awareness and knowledge among GPs about the CVR of APM, and it facilitated the start of regular monitoring.

In this feasibility study, all patients taking APM were approached by the GP. HCPs stated that a barrier, owing to approaching as many people as possible, is that you include the ‘*worried well*’. Both patients and HCPs found it important to establish a well-defined indication for TACTIC, where TACTIC should aim at patients who can really benefit from it. Since the goal of TACTIC is decreasing CVR, they suggested that TACTIC should target patients with a relatively high risk.

Both patients and HCPs suggested that it should be more clear that the goal of TACTIC is not tapering off or stopping APM per se, but adjusting where necessary and trying to reduce CVR where possible, taken into account the personal needs of the individual patient:

*‘Before you go into the whole process, hey, if it’s going to become a treatment method, estimate very carefully whether it really adds value for the patient in question*.’ (P03)

HCPs also stressed that the MDM should focus on the topic and should not be used to review a whole case or diagnosis.

### Expectations of the intervention

Before the intervention, patients’ expectations varied. Some merely wanted to discuss their side effects, and saw TACTIC as an excellent opportunity. Others expected to find more answers on their specific, personal questions. They felt a barrier in having to explain their problems and elaborate their questions to the HCPs.

HCPs noticed another barrier: their expectations did not always match those of the patients. Before the MDM, the objective of the meeting should be more clear. It was suggested that before the intervention, it should be specifically recorded what the patient wants to achieve. Sometimes it only became clear during the MDM what the patient’s question was:

*‘… maybe check at the beginning: this is what we want to talk about, what do you want to talk about? Otherwise there will be another one of those jack-in-the-box things at the end*.’ (GP5)

### Experiences and feelings

Participating in TACTIC created tension in some patients. They were reluctant to change their medication, to let go of the certainty they experienced with their APM. They were afraid of relapsing or having a crisis. Because of this tension, they experienced a great amount of stress during the MDM, which was a barrier for active participation:

*‘I found it very stressful to participate. Because yes that ... It is drastic in your life when you are going to change something. I found that very stressful*.’ (P06) *‘He actually experienced it all very emotionally. From his own fear of: I can’t do without antipsychotics*.’ (C06)

Several patients reported that their needs and ideas were addressed at the MDM. They experienced the MDM as pleasant. Others, however, perceived the setting of the MDM as a barrier as the amount of attendants was overwhelming and the MDM was mainly a conversation between the patient and the psychiatrist. Some patients experienced little openness to their choice of not wanting to change medication. They sometimes felt unheard.

The SDM visit was experienced as very pleasant by patients. Not all advice was immediately turned into action, but it was now open on the table, which facilitated consideration at a later date.

During the feasibility study, there was no limit in including patients, hence all patients taking APM were approached. This led to GPs experiencing a high workload, which felt as a great barrier for their engagement. HCPs expressed the opinion that the success of TACTIC depends on the level of CVR, but also on the patient’s motivation. They considered TACTIC most useful for chronic APM users and patients with high CVR and suggest to specifically target these patients:

*‘When you talk about selection etc., it’s about the goal you're aiming for. Are you aiming mainly to start seeing or working with patients who have an increased cardiovascular risk* [...] *then you could say: that is the category that is most relevant to look at critically*.’ (Ps)

### Communication and information

A facilitating factor in delivering the intervention was the information presented during the information meeting. Both HCPs and patients experienced it as a good preparation for the MDM. It facilitated in raising the right expectations and allowed the participating HCPs to introduce themselves. Patients regarded the information meeting as clear, but the information provided during invitation was, to some, already sufficient. From a pragmatic perspective, it was suggested that the webinar could be replaced by an information video:

*‘What I can imagine is just some kind of standard video in terms of, say, the people participating in this project*.’ (Ps)

Information about CVD prevention was considered a facilitating factor by both patients and HCPs. This was also highlighted during TACTIC, but according to some patients, insufficiently.

While several patients reported that the attendants of the MDM were well informed, others expressed the opinion that the attending HCPs had not adequately reviewed their case before the MDM, particularly the psychiatrist. To them, this was perceived as a barrier during the MDM.

Without being asked, the psychiatrist wrote a detailed summary of their advice given during the MDM, to be used by the GP, the practice nurse, and the patient during the SDM visit and further follow-up. This was a well-appreciated facilitating factor by both patients and HCPs. With this summary, they had an overview of what had been discussed in the MDM. It gave them something to hold on to, something to read again, because patients reported missing an evaluation with the psychiatrist during follow-up as a barrier for success:

*‘I read them all, also as a piece of preparation when people came to me after 3 months. I did enjoy reading them. I also put them neatly in the medical record, absolutely great*.’ (CN1)

### The role of HCPs

The invitation of patients for TACTIC by the GP took a lot of time and effort and was perceived as an important barrier. However, GPs indicated that they consider personally calling and inviting patients, although very time-consuming, to be the most effective. The degree of trust established between a GP and the patient is an important facilitator in this context, and it is not desirable to outsource this, according to the GPs, despite the high workload:

*‘It’s the energy it took and the time it took and the yield, those were not feasible for me either*.’ (GP5)

*‘If the GP calls themselves, that does help, yes. That’s pretty labour intensive, I do think, for the GP in this case, but it is the most effective way to include patients, I think*.’ (GP1)

The fact that TACTIC is multidisciplinary was being encouraged. Patients found it valuable that their individual situation was being looked at from different angles as this facilitated collaboration between different disciplines.

Patients were free to bring a carer and had the opportunity to determine whether the expert with lived experience would be present at the MDM. The ability to adjust the roles during the intervention was appreciated by the patients. It facilitated participation and gave them a sense of control.

It was found important by both patients and HCPs to attract the right professionals, tailored for the patient, where equality of roles within the MDM deserved attention.

According to some patients, the MDM could have been performed on a smaller scale, with only the patient, the GP, and the psychiatrist attending. The chronic care nurse, however, valued her participation in the MDM. This way, she had already met the patients, which facilitated monitoring them during follow-up.

The presence of the expert with lived experience was appreciated by both patients and HCPs. Because participation can be quite stressful, HCPs saw a facilitating role for the expert as ‘*sidekick’* for the patient. The role of the expert, however, did not always fulfil its potential. A perceived barrier was the lack of clarity regarding the expert’s role in the broader context of the intervention. The expert with lived experience mainly had value when they matched the patients in terms of problems.

Both patients and HCPs believed it was important to deploy an expert with adequate training and experience.

The few patients who brought a carer appreciated their attendance. Furthermore, the carer saw a facilitating role for themselves. However, many patients saw no benefit in bringing a carer.

The added value of the medication review before the MDM was questioned by both the pharmacist and the GPs. It was perceived as a barrier that the pharmacist executed the medication review on paper. Without a complete picture of the patient, they could only provide general advice. The pharmacist suggested to attend the MDM themselves, so that their input could be more useful. They felt that their advice may contribute to better treatment compliance when they speak directly with the patient. Consequently, increased patient’s involvement may increase treatment success. However, GPs felt that a pharmacist would not belong in the MDM, and their absence would also prevent the MDM from becoming overcrowded. Instead, they suggested a personal review between GP and pharmacist before the MDM, to inform the GP with proper advice.

## Discussion

### Summary

This feasibility study revealed the following three main barriers for delivering TACTIC: the tension towards the intervention experienced by patients; the course of the MDM; and the high workload for the GPs. Main facilitators were the person-centred approach, the clear information meeting, and the ability of adjusting the roles during the course of the intervention, including bringing a carer. We identified valuable suggestions for improvement of the intervention among which are adjustments to the definition of the right target group, improving expectation management for patients, and introducing a summary report of the MDM from the psychiatrist. These findings are promising and show that, with some modifications, TACTIC is ready for evaluation in a large RCT. In Table 4 we present an overview of the adaptations we made in the procedures of the upcoming trial.

**Table 4.** Adaptation to trial procedures

Problem	Solution
GPs spent too much time recruiting patients	Provide better support for GPs during recruitment
High workload at research sites	Maximise the number of patients per practice
Patients feared they would have to stop antipsychotics	Clearly state in patient information that discontinuation is not mandatory
Patients were insufficiently prepared for the MDM	Focus baseline consultation on patient goals; offer a consultation with a pharmacist or expert with lived experience
HCPs were insufficiently prepared for the MDM	Share patient expectations via the electronic medical record
Missing data on prior antipsychotic use and mental health care	Add a patient questionnaire to capture medical history
Information meetings or webinars were not feasible for large-scale implementation	Replace webinars with information videos featuring the patient’s healthcare team
Need for a clear summary of advice	Request a report from the psychiatrist suitable for the GP, chronic care nurse, and patient

HCPs = healthcare professionals. MDM = multidisciplinary meeting

### Strengths and limitations

This study provides valuable insights into the experiences and beliefs of patients and HCPs after implementation of a new person-centred collaborative care intervention for patients on APM.

As far as we know, this is the first study to evaluate barriers and facilitators involved in delivering a multidisciplinary intervention specifically aimed at lowering CVR in patients on APM, both with and without SMI, in general practice.

The topic guides were developed iteratively: each interview led to adjustments and was adapted to the situation. This led to increasingly rich data. After six patient interviews, data saturation appeared to have occurred, which was confirmed after conducting two additional interviews. This suggests that there was enough information gained to soundly answer our research questions.

To select patients for this qualitative study, we purposively sampled them. However, we only selected patients who completed the entire study and we were only able to interview those who we were able to reach and who were willing to participate. Perhaps patients who were not available for interviewing would provide other information than the patients we did interview. We have tried to facilitate participation in the interviews as much as possible by giving patients the choice of how, where, and when the interviews took place. Furthermore, since the vast majority of reasons of not completing the entire study was unrelated to the intervention, as reported by Jakobs *et al*,^35^ the probability of highly divergent answers is low. Some selection bias, however, cannot be completely ruled out.

Owing to COVID-19 measures, the focus group interviews could not take place physically. We were forced to hold them via video calling. Thanks to good instructions (for example, everyone left their camera and microphone on, so that there was no obstacle to saying something), we tried to imitate a real-group setting as much as possible. However, responding to non-verbal communication, such as gestures or facial expressions, is more difficult via video calling than in real life.^45^ The advantage of focus groups via video calling was that all participants were recorded both as a group and individually, so that no information was lost when, for example, participants talked over each other.

As explained in the methods section, we chose to organise and present our findings according to the emerged themes, instead of using the NPT constructs, with the limitation that we did not use a theoretical framework in the analysis. We did use the NPT to inform our research questions and topic guides. However, themes appeared too comprehensive to fit them into the predetermined constructs of the NPT, so we chose to present them in the same way as we analysed them. This enabled us to use all valuable data derived from the interviews.

### Comparison with existing literature

In our study, GPs experienced a high workload, which was partly attributable to patients being difficult to reach or failing to attend scheduled appointments. Literature shows that patients with an SMI have difficulty attending appointments,^46^ owing to various reasons, associated with poverty, unstable housing, or side effects of mediation.^40^ Other barriers can be motivation and adherence to treatment. It is crucial to consider these elements, as they are the very factors that can render CVD risk-lowering strategies more challenging in this target group.^39^ Deployment of experts with lived experience could help to involve patients more, but also involving supportive others is a possible strategy to make CVR management more successful.^39^ These experts with lived experience and carers can help managing appointments, or even accompany patients to appointments.^40^ In our study, relatively few carers were involved, and their value was viewed variably.

Patients mentioned that they were reluctant to change their medication. They were afraid of relapsing or having a crisis. This perceived tension had an impact on their participation in TACTIC. Crawford *et al* also encountered this high level of concern, even leading to a low level of recruitment for their trial.^47^ Patients who feel secure with their medication and/or experience fewer side effects may be more reluctant to change.^47,48^ Since switching APM may increase the risk of relapse^49^ or cause new side effects,^50^ patients’ concerns are not unfounded. However, during TACTIC, medication is only changed on the advice of an experienced psychiatrist, when this is a safe option only, and always through SDM. Patients must receive this reassurance in advance clearly, in order to facilitate participation in TACTIC.

This underscores the importance of proper expectation management, but also of the person-centred approach in TACTIC. Seen as a promoting factor for the intervention, personal and continuous care^51–53^ is important for enrolling and retaining patients from a more or less vulnerable target group, as are patients in TACTIC.

As mentioned during the interviews, regarding to the target group, it is important to establish a proper indication for TACTIC, meaning that TACTIC should not just focus on *every* patient on APM, but merely on those with a relevant risk of developing CVD. There are various risk calculations for CVR in patients. In the Netherlands, SCORE2 is widely used in general practice,^31^ while in England QRISK3 is used.^21^ In contrast with SCORE2, QRISK3 also includes the use of APM and having an SMI in the 10-year risk calculation.^21^ In view of the population of our interest, QRISK3 seems more appropriate to use in TACTIC. Introducing a threshold of a 5% 10-year risk for inclusion seems reasonable to the authors, as it would decrease the workload for GPs and hence benefit the feasibility of TACTIC.^35^ Especially in young people with psychosis, the risk of developing CVD may be underpredicted with QRISK3.^54^ Inviting these relatively young patients with a relatively low CVR will start a GP–patient relationship enabling follow-up and early detection when risk factors, such as overweight, do become apparent. Choosing a higher threshold of 10%, as is used in the UK National Institute for Health and Care Excellence (NICE) guideline on cardiovascular disease risk assessment and reduction,^55^ would have the undesirable effect of excluding a large group of mainly young patients, for they generally do not reach 10%.^35^

### Implications for research and practice

This study yielded valuable lessons for redesigning and improving the TACTIC intervention. We combined both focus group and individual interviews, addressing the strengths of both methods, and were able to conduct sufficient individual interviews in a hard-to-reach target group by adapting to their circumstances.

With the intention of eventually implementing TACTIC in standard practice, it is important to reduce the workload in deploying the intervention and increase its yield. The study findings do not only inform us in adjusting the intervention for further evaluation, but also they are useful for other researchers developing complex interventions, especially in hard-to-reach target groups. The results highlight the valuation of a personal and person-centred approach in relatively vulnerable patients, and the ability of adjusting roles, tailored to the patient. Our results underscore the importance of good expectation management, especially in a relatively vulnerable target group, as tension during an intervention may influence effectiveness. Defining the appropriate target group for an intervention will also improve efficacy.

All things considered, the analysis emphasises the importance of performing a qualitative analysis as part of a mixed-methods feasibility study before a larger trial when developing a complex intervention, as this approach is likely to enhance its effectiveness and efficacy.

With the right adjustments, TACTIC is ready to be evaluated in a large RCT, with which the effectiveness and implementability can be further investigated.

## Data

The dataset relied on in this article is available from the corresponding author on reasonable request
